# Toll-Like Receptors Promote Mutually Beneficial Commensal-Host Interactions

**DOI:** 10.1371/journal.ppat.1002785

**Published:** 2012-07-26

**Authors:** Jason L. Kubinak, June L. Round

**Affiliations:** Department of Pathology, Division of Microbiology and Immunology, University of Utah School of Medicine, Salt Lake City, Utah, United States of America; The University of North Carolina at Chapel Hill, United States of America

Toll-like receptors (TLRs) are evolutionarily conserved trans-membrane receptors that detect micro-organisms through the recognition of conserved molecular motifs. With each TLR able to recognize a discrete set of microbial ligands, TLRs represent an important mechanism by which the host detects a variety of microorganisms. Triggering of TLRs by pathogens is classically understood to result in an inflammatory immune response that supports the subsequent clearance of the offending microbe. However, animals are stably colonized by a consortium of symbiotic microbes (the microbiota) that possess many of the same structural motifs as pathogens, which raises the question of how the host is able to peacefully coexist with this community of organisms without suffering from chronic inflammatory disease. Paradoxical to its pro-inflammatory role during infection, recent studies have suggested that commensal bacterial recognition by TLRs is important for limiting inflammation within the intestine. While TLR expression on cells of the innate immune system (macrophages and dendritic cells) is largely thought to account for TLR function, cells of the adaptive immune system (T and B cells) and non-hematopoietic (epithelial) cells also express TLRs. Recent evidence has uncovered that the functional consequence of TLR engagement depends largely on the responding cell type and does not always result in inflammation. Here we will highlight how TLR engagement on epithelia, B and T cells promotes mutually beneficial interactions between hosts and their resident microbes.

## Expression of TLRs within the Intestine

**Figure 1:**
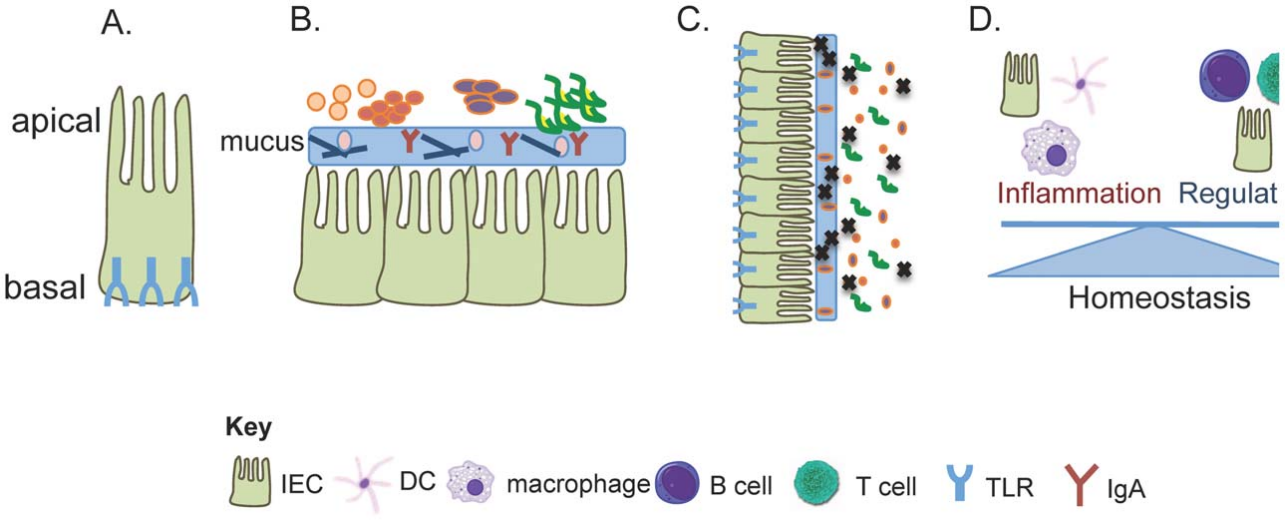
TLR signaling controls multiple pathways for tolerating the commensal microbiota. Crosstalk between the immune system and the microbiota can limit disease in three non-mutually exclusive ways; by limiting the migration of microbes into sensitive host tissues by promoting healthy barrier function (i.e. spatial segregation), by changing the community of organisms that colonize the host (some may be disease protective while others may be disease inductive), and by educating the immune system to be tolerant of innocuous members of the microbial community. (A) Intestinal epithelial cells are the first cells to come into contact with luminal bacteria. Multiple studies have demonstrated that a mechanism to avoid overt inflammation toward the intestinal bacteria is to sequester TLRs toward the basal compartment of the IEC. Therefore, TLRs, and subsequently inflammation, are only engaged when bacteria have penetrated host tissue. This represents a passive mechanism to avoid chronic inflammation. (B) Spatial segregation of the commensal microbiota is another mechanism to avoid inflammation within the intestine. A thick, organized mucus layer that is composed of anti-bacterial proteins, mucins, and antibodies creates a barrier between the host tissue and the luminal bacteria. (C) Several examples exist that demonstrate how TLR signaling can influence the diversity of the microbial community. Additionally, a single commensal species, *B. fragilis*, has been shown to utilize TLR2 signaling to colonize the host tissue. Therefore, a mutation in any one of the TLRs could lead to a loss of colonization by a beneficial commensal and a change in the structure of the microbial community. Changes in microbial community dynamics could lead to a loss of tolerance within the intestine. (D) It is now becoming appreciated that cells of both the innate and adaptive immune system have functional TLR receptors. TLR signaling on various cell types can have differential functional consequences. Indeed, while triggering of TLR signaling on macrophages has long been known to induce inflammation, it was just recently demonstrated that B cell-intrinsic TLR signaling is important for maintaining tolerance within the intestine. Therefore, TLRs represent a way in which the commensal microbiota can communicate with the host and loss of this machinery could disrupt intestinal tolerance.

The intestinal epithelium provides a physical barrier between the luminal contents of the gut and underlying host tissues. Importantly, this tissue also represents the major site of crosstalk between the immune system and the microbiota. Intestinal epithelial cells (IECs) are both structurally and functionally polarized, with the apical surface of the cell facing the intestinal lumen and the basolateral surface in contact with the lamina propria [1]. TLRs are expressed at variable levels within both the human and mouse intestine, and multiple TLRs are upregulated in response to commensal colonization [2]. TLR expression within IECs is spatially regulated with expression being generally restricted to the basolateral surface of IECs or sequestered within the cell (Figure 1A) (reviewed in [1]). This mechanism ensures that an immune response is only mounted when bacteria penetrate the host epithelial layer. Also, the functional consequence of TLR signaling depends on the site of the cell where the ligand is encountered. While TLR9 is expressed on the apical surface of IECs, inflammatory signaling only occurs when basolaterally expressed TLR9 interacts with its appropriate ligand [3]. Finally, negative regulators of TLR signaling, such as Toll-interacting protein (TOLLIP), are preferentially expressed in IECs and represent another mechanism employed by IECs to create a tolerant environment within the intestine. Taken together, regulating expression of TLRs and sequestering these receptors from chronic stimulation by the microbiota represent a passive mechanism to ensure tolerant reactions to the commensal microbiota. The rest of this review will focus more specifically on how TLR signaling actively maintains tolerant interactions between the host and the microbiota.

## TLRs Instruct Spatial Segregation of the Microbiota

The physical barrier between the lumen and underlying host tissues created by the intestinal epithelium is enhanced by the presence of a thick layer of mucus overlaying the apical surface of IECs [4]. One of the most important features of the mucus layer is to create spatial segregation between the host and the commensal bacteria (Figure 1B). Major constituents of the mucus include the highly glycosylated MUC2 mucin and anti-bacterial proteins such as RegIIIγ, IgA, and IgM. The mucus is an organized structure that contains both a dense area that is in direct contact with the epithelial lining and a loose outer layer. The outer layer has an expanded volume as it is exposed to proteolytic activities from both the host and the commensal microbiota [5], [6]. While bacteria can be seen inhabiting the outer layer of mucus, the denser level is largely devoid of commensals [6].

Germ-free mice still possess a mucus layer, demonstrating that the production of mucus is not dependent on the presence of commensals [5]. However, in the absence of the microbiota, critical components of the mucus layer are lacking, such as RegIIIγ [7], [8]. Recent data have demonstrated that sensing of commensal bacteria by TLRs is important for spatial segregation of the microbiota and the production of RegIIIγ [8], [9]. Indeed, in MyD88−/− animals, which lack most TLR signaling pathways, there is a complete loss of host-commensal separation such that most of the microbiota is in direct contact with IECs [8]. More importantly, MyD88 signaling within IECs themselves was responsible for this activity. Supporting this, mice deficient in RegIIIγ also have a defect in spatial separation, demonstrating the importance of these anti-microbial proteins to mucus function. Therefore, host IECs respond directly to the commensal microbiota through TLRs and consequently increase the production of anti-bacterial proteins that enhance barrier function.

Cells of the adaptive immune system have also been implicated in sustaining barrier integrity. Eighty percent of all B-cells in the body are found within the intestinal lamina propria where they produce more IgA than any other antibody class combined [10]. IgA and IgM are major constituents of the intestinal mucus layer that help to maintain spatial segregation of the microbiota. The presence of these antibodies is dependent on the microbiota [11], [12]. Recently, TLR signaling in B-cells was shown to be important for maintaining barrier function. Indeed, animals lacking B-cell-intrinsic Myd88 expression had reduced IgM and complement expression that resulted in dissemination of commensal bacteria into the systemic compartment [13]. As MyD88−/− mice do not develop spontaneous intestinal disease [14], it seems that maintaining spatial segregation of the microbiota is not detrimental to the host during the steady state. However, upon challenge with a model of innate immune colitis, MyD88−/− mice succumb more quickly to inflammatory disease [13]. Therefore, TLR-mediated detection of the commensal microbiota by B cells and IECs facilitates spatial segregation of host and microbe and represents an important mechanism by which tolerance of the microbiota is achieved.

## TLR Signaling Coordinates the Composition of the Microbiota

Multiple studies have compared the microbial communities between healthy individuals and those with inflammatory bowel disease (IBD). Interestingly, while healthy individuals tend to have species from the phyla *Firmicutes* or *Bacteroidetes* as the dominant microbial community within the gut, individuals with IBD have a completely different representation of microbes [15]. These data have led to the notion that the composition of the microbial community may play an important role in the maintenance of intestinal homeostasis and host health [16]. Studies have now demonstrated that TLR signaling can influence the structure of the microbial community (Figure 1C). A lack of MyD88 signaling in some mouse strains significantly changes the composition of the microbiota and this altered microbial community predisposes the host to disease [17]. An example of this is seen in TLR5 knockout mice, which develop spontaneous colitis and metabolic abnormalities in some mouse colonies. Induction of disease was associated with significant shifts in the composition of the microbiota [18]. Transferring the microbiota from diseased TLR5−/− animals into germ-free wild-type mice reproduced the observed metabolic disease phenotype. Similarly, TLR2−/− animals develop insulin resistance that can be induced in germfree wild-type mice by simply transferring the microbiota from a TLR2−/− animal [19]. These data support that TLR sensing of the commensal microbiota can shape the community, but it is unclear how this occurs. A clue was provided by studies performed in germ-free animals that were mono-associated with the commensal organism, *Bacteriodes fragilis*. While most bacteria are kept away from the epithelium, a small population of *B. fragilis* lies in close proximity to the host tissue [20]. In the absence of T-cell-intrinsic TLR2 signaling, *B. fragilis* could no longer reside within this intestinal niche and was instead restricted to the lumen. The ability of this organism to signal through TLR2 relies on production of a surface polysaccharide called PSA. Engagement of TLR2 by PSA uniquely suppressed host inflammatory responses that allowed for the commensal to persist on host tissues. Therefore, commensal organisms may have evolved specialized molecules that exploit TLR signaling in order to colonize the host. Consequently, this represents a potential mechanism by which TLRs determine the composition of the microbiota, as mutations in TLRs or their signaling pathways could prevent certain microorganisms from residing within the intestine.

## Commensal Manipulation of TLR Signaling Induces Tolerant Responses

Preserving a balance between inflammation and regulation within every tissue is paramount to sustaining health. Overstimulation of anti-inflammatory pathways would leave the host susceptible to pathogenic infection, while chronic inflammation leads to severe autoimmunity. Indeed, diseases such as IBD, diabetes, multiple sclerosis (MS), and arthritis result from the constant stimulation of inflammatory pathways. Ironically, despite their established role as instigators of pro-inflammatory immune responses, studies are uncovering a role for TLRs in preventing intestinal inflammatory disease. Indeed, upon challenge with experimental models of IBD, MyD88−/− animals develop more severe intestinal inflammation than wild-type mice, despite the complete lack of TLR signaling [14]. More importantly, B-cell-intrinsic MyD88 signaling is important for protection from experimental IBD [13]. Using multiple MyD88 tissue-specific knockout strains, it was shown that only when B cells lacked TLR signaling did the animals succumb to severe disease. As mentioned earlier, protection from disease by B cells is conferred by enhanced barrier function. Therefore, it seems that TLR signaling represents an important component in maintaining homeostasis.

There are several examples of commensal bacterial molecules exploiting TLR signaling to inhibit inflammation. *B. fragilis* was mentioned earlier for utilizing the TLR2 pathway to persist on host epithelia. Interestingly, the same mechanism that *B. fragilis* employs to colonize host tissue also protects the host from autoimmunity. Colonization of mice with *B. fragilis* protects animals from developing experimental IBD and this protection is dependent on TLR2 [21], [22]. Upon administration of purified PSA to animals, an anti-inflammatory population of T regulatory cells (Tregs) is induced within the intestine. Interestingly, PSA signals directly to T cells through TLR2 to induce this regulatory response, again highlighting that adaptive immune cells can directly respond to commensals through TLRs [20], [23]. Other organisms apply similar strategies to protect the host skin from damage. *Staphylococcus epidermidis* is a resident of skin that does not typically elicit inflammation. A small molecule secreted by *S. epidermidis* blocks inflammation in keratinocytes during a response to double-stranded RNA [23]. The ability of this small molecule to control inflammation was dependent on TLR2 signaling, providing another example of how TLR signaling can induce anti-inflammatory responses through sensing of commensal bacteria.

Collectively these data highlight the importance of TLR signaling during host-commensal associations. Intrinsic TLR signaling in cells of the adaptive immune system represents a potentially important development in our understanding of how the host immune system elicits tolerant responses to commensal organisms. In turn, commensals have evolved strategies to manipulate TLR pathways that under some circumstances can protect hosts from inflammatory disease. Therefore, TLR signaling is paramount not only for protection from pathogenic infection, but also for inducing tolerant responses to commensals. Understanding how commensals exploit differential TLR signaling pathways and how TLRs influence the function of adaptive immune cells will allow for a better understanding of how the host is able to discriminate “friend” from “foe.”
